# Supplementary material for: FOXE1-Dependent Regulation of Macrophage Chemotaxis by Thyroid Cells In Vitro and In Vivo
Source: Int J Mol Sci. 2021 Jul 17;22(14):7666. doi: 10.3390/ijms22147666 (PMC8307198; doi:10.3390/ijms22147666)
Supplement: Supplementary file 1 [file ijms-22-07666-s001.zip › ijms-1275731 supplementary 2.pdf]

A

Gene Symbol	Fold change FC55 vs FRT	p-value
ABLIM	3.68	2.43e-03
IFI204	4.09	3.72e-18
SOX6	3.16	9.72e-04
CELSR2	4.24	4.10e-246
RHOQ	3.04	3.93e-103
CLSTN3	3.66	8.10e-03
CFH	1197.50	8.53e-64
LCP1	337.86	0.00e+00
UNC13D	-4.78	1.26e-11
DPP4	-3.64	7.08e-108
CAV1	20.73	1.19e-03
TAC4	-3.35	4.73e-02
SLC1A2	14.36	3.85e-21
HEPH	1673.75	1.44e-180
ADM	5.61	2.03e-06

B

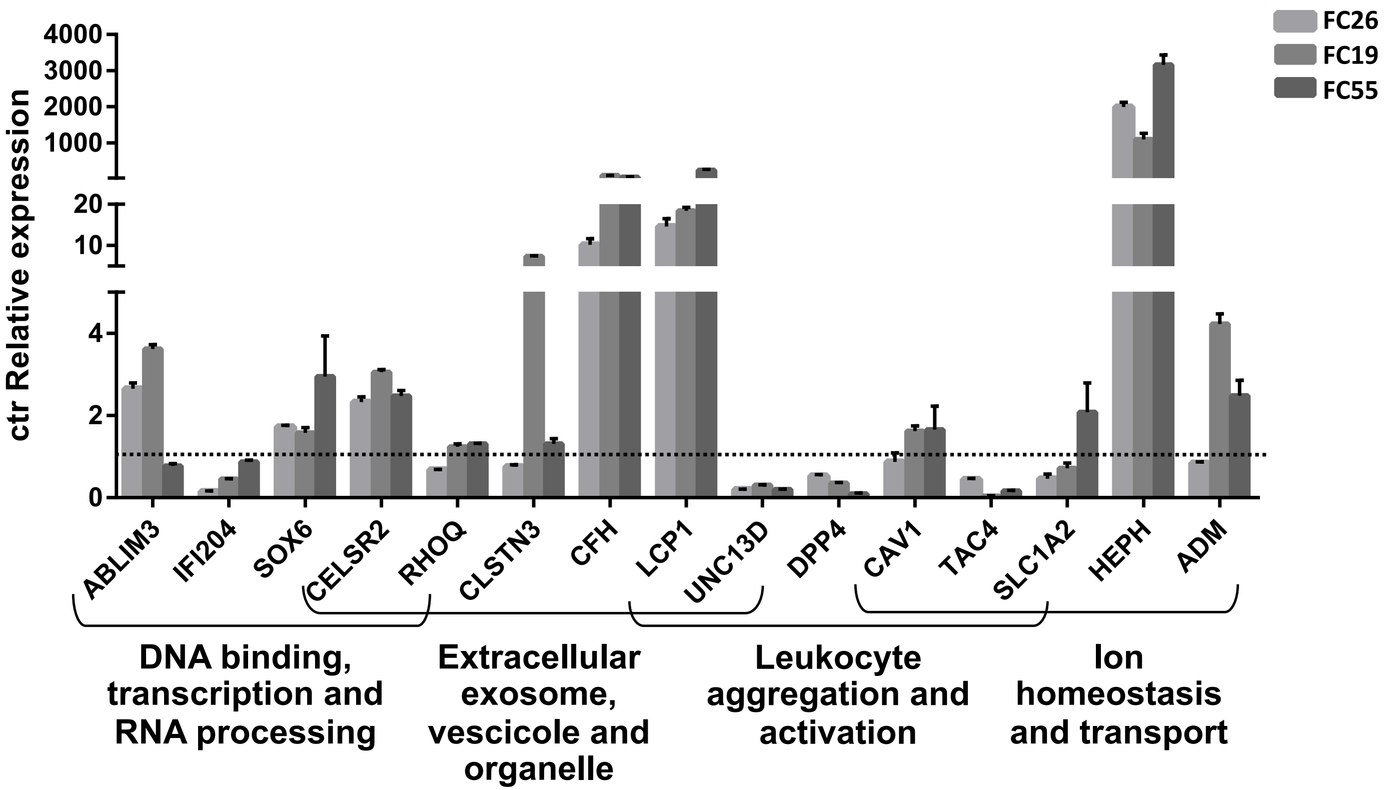

Figure S1: Validation of DEG in FRT-overexpressing clones. A. Selected genes from the results of RNA-seq performed on *FOXE1* overexpressing CF55. **B.** A qrt-PCR analisis, for the genes in A, was performed on *FOXE1*-clones compared to control FRT cells (ctr). The data are shown as ctr-relative expression (2^-ΔΔCt) and reported referring to the functional category they belong to. The dotted line represents the ctr value set to 1. *TUBULIN* was used to normalize.

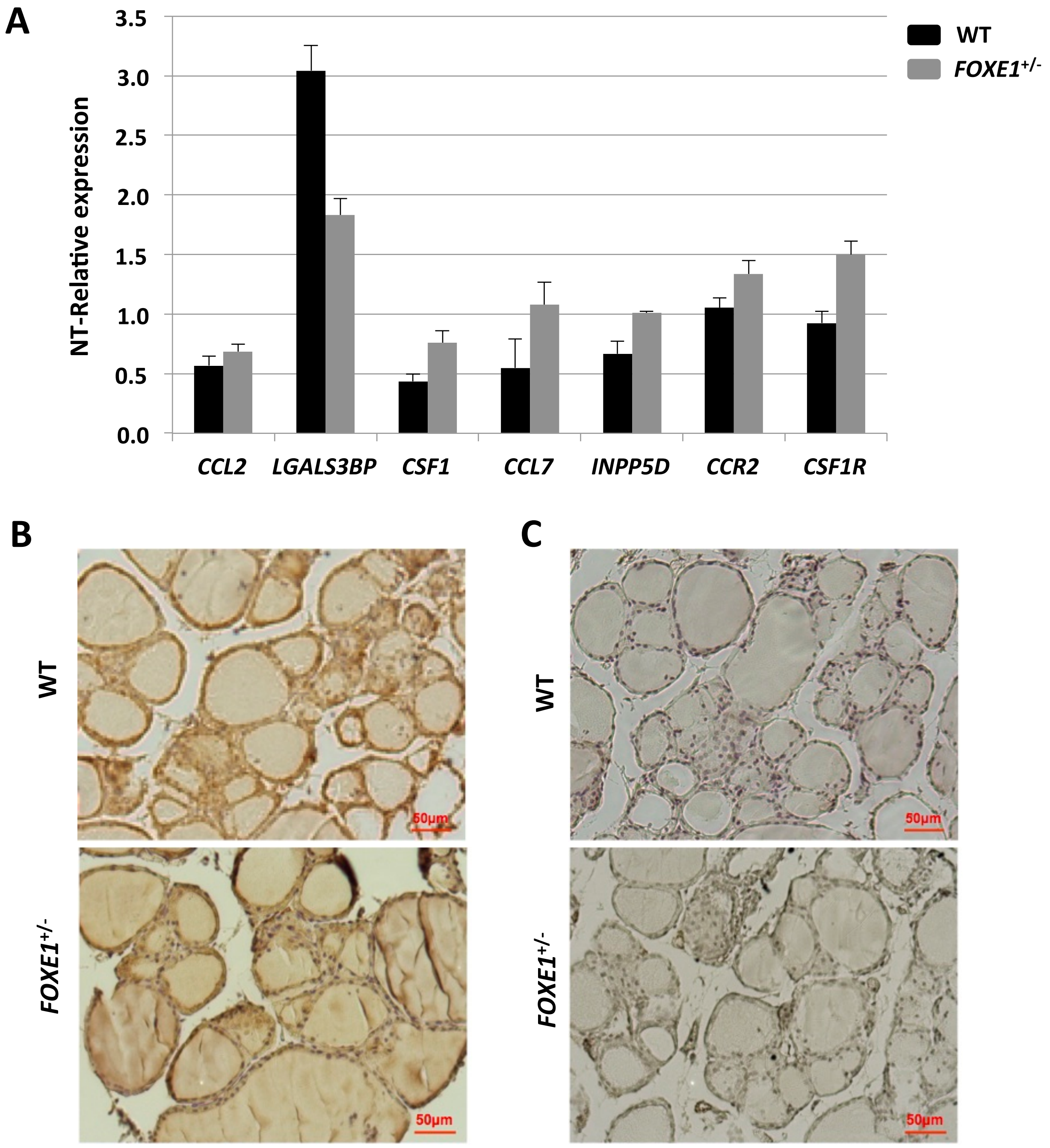

Figure S2: A. Total RNA was extracted from thyroids of doxycycline treated and untreated (NT) WT and *Foxe1*$^{+/-}$ mice and macrophages related markers were analyzed by quantitative RT-PCR. Results are reported as the fold change (2^-ΔΔC_t) of WT and *Foxe1*$^{+/-}$ treated mice respect to the corresponding untreated mice. Means ± SD are shown. T-Test was performed on data founding no significance. **B, C.** IHC staining for IBA1 (B) and GALECTIN-3 (C) was performed on 7μm section of thyroids from WT and *Foxe1*$^{+/-}$ mice after doxycycline treatment. Hematoxylin was used to stain the nuclei. 20X magnifications are shown. Each image is representative of six different images.
